# Maximising response from GPs to questionnaire surveys: do length or incentives make a difference?

**DOI:** 10.1186/1471-2288-15-3

**Published:** 2015-01-06

**Authors:** Elizabeth Cottrell, Edward Roddy, Trishna Rathod, Elaine Thomas, Mark Porcheret, Nadine E Foster

**Affiliations:** Research Institute for Primary Care & Health Sciences, Keele University, Keele, Staffordshire ST5 5BG UK

**Keywords:** Cross-sectional survey, Postal questionnaires, General practice, Incentive, Non-response, Questionnaire length, Response

## Abstract

**Background:**

General Practitioners (GPs) respond poorly to postal surveys. Consequently there is potential for reduced data quality and bias in the findings. In general population surveys, response to postal questionnaires may be improved by reducing their length and offering incentives. The aim of this study was to investigate whether questionnaire length and/or the offer of an incentive improves the response of GPs to a postal questionnaire survey.

**Methods:**

A postal questionnaire survey was sent to 800 UK GPs randomly selected from Binley’s database; a database containing contact details of professionals working in UK general practices. The random sample of GPs was assigned to one of four groups of 200, each receiving a different questionnaire, either a standard (eight sides of A4) or an abbreviated (four sides of A4) questionnaire, with or without the offer of an incentive (a prize draw entry for a £100 voucher) for completion. The effects of questionnaire length and offer of incentive on response were calculated.

**Results:**

Of 800 mailed questionnaires, 19 GPs did not meet inclusion criteria and 172 (adjusted response 22.0%) completed questionnaires were received. Among the four groups, response ranged from 20.1% (standard questionnaire with no incentive and abbreviated questionnaire with incentive) through 21.8% (standard questionnaire with incentive), to 26.0% (abbreviated questionnaire with no incentive). There were no significant differences in response between the four groups (p = 0.447), between the groups receiving the standard versus the abbreviated questionnaire (% difference -2.1% (95% confidence interval (CI) -7.9, 3.7)) or the groups offered an incentive versus no incentive (% difference -2.1% (95% CI -7.9, 3.7).

**Conclusions:**

Strategies known to improve response to postal questionnaire surveys in the general population do not significantly improve the response to postal questionnaire surveys among GPs. Further refinements to these strategies, or more novel strategies, aimed at increasing response specifically among GPs need to be identified in order to maximise data quality and generalisability of research results.

**Electronic supplementary material:**

The online version of this article (doi:10.1186/1471-2288-15-3) contains supplementary material, which is available to authorized users.

## Background

The cross-sectional postal questionnaire survey is a valuable research method which allows large sample sizes to be mailed to gather data efficiently and relatively cheaply, using a consistent stimulus, at a single time-point. This approach also allows participants to respond to the survey at a time that is most convenient to them. However, response from General Practitioners (GPs) to postal questionnaire surveys is notoriously poor [1, 2]. A review of published GP surveys identified a mean response of 61% (95% confidence interval (CI) 59, 63), but showed higher levels of response among journals with higher impact factors and a declining response over time [3]. GP surveys often fail to obtain response above 50% [4–6]. Low response risks response bias and thus threatens the generalisability of results obtained [7–10]. Strategies such as following-up questionnaires with telephone calls and/or face-to-face visits have resulted in improved response from GPs [2, 10–13], yet these strategies may not be feasible with large sample sizes and may represent a significant burden to participants.

Response among a broad range of populations, including patient and general populations, and healthcare and non-healthcare professionals, can be improved by reducing the length of the survey [14] and using financial incentives [14, 15]. However, few studies have specifically tested these strategies with GPs and, among published GP questionnaire surveys, reporting of methodologies is often incomplete [3]. It is possible that empirical research findings from more general populations may not be directly transferrable to GPs, who may receive multiple requests to complete surveys each year and who experience significant time and workload pressures [9, 12, 16]. To maximise participation in survey research, questionnaires must be sufficiently short and incentives large enough to affect response. What constitutes an ‘appropriate’ length and/or incentive is uncertain [14, 17]. The aim of this study was to investigate whether questionnaire length and/or the offer of an incentive (a prize draw entry for a £100 voucher) improved response of GPs to a postal questionnaire survey.

## Methods

This survey addressed one of the primary aims of a national pilot study that was undertaken to inform a planned main survey, to investigate the attitudes, beliefs and reported clinical management of GPs regarding exercise for chronic knee pain (CKP). Completion and return of the questionnaire survey by the GP was taken as consent to participate in the study. Ethical approval for this study was obtained from Keele University Ethical Review Panel.

A postal questionnaire survey was sent to a random sample of UK GPs obtained from Binley’s Database; a database containing the contact details of professionals working in UK GP practices which is updated quarterly. To achieve a balance between minimising burden on GPs and achieving a good estimate for the outcomes, a response target of 200 completed questionnaires (i.e. 100 of each version [18]) was desired. Expecting a response of approximately 25% [4], 800 GPs were surveyed. Each GP in the sample was given an ID number from 1–800 according to the random order in which they were supplied. GPs were allocated to one of four groups according to their ID number:

1. GPs 1–200 were sent a standard questionnaire (StQ) and offered an incentive (see Additional file 1), a prize draw entry for a £100 voucher.

2. GPs 201–400 were sent a StQ with no incentive offered.
3. GPs 401–600 were sent an abbreviated questionnaire (AbQ) and offered an incentive.
4. GPs 601–800 were sent an AbQ with no incentive offered (see Additional file 2)

The StQ was eight sides of A4 in length, contained 85 items, and took 15 minutes to complete. To address the aims of the planned main survey, the StQ collected information about a) demographic characteristics (gender, year of qualification, practice size and setting, type of GP, GP with special interest in musculoskeletal conditions, previous under/postgraduate training in CKP, personal experience of CKP), b) the GPs’ views about the cause and nature of CKP, c) the GPs’ investigation and management of a vignette patient, d) use of exercise for CKP and 5) familiarity and attitudes about best practice guidelines.

The questionnaire was presented in an A4 booklet format, created from folded and stapled A3 pages. Therefore to make a clearly tangible difference in length (i.e. to reduce the number of pages, not just the number of questions) the length of the AbQ was designed to be four sides of A4. The AbQ, contained 36 items and took 10 minutes to complete. The AbQ was created by abbreviating all sections of the StQ except section d), which remained the same. The items, used in the AbQ, were identical to those in the StQ except in two cases: two closed multiple response items in section c) of the StQ, regarding investigation and management of the vignette patient, were reworded as open, free text, questions in the AbQ. The questionnaires were printed as booklets on white paper with the institution logo on the front cover.

The financial incentive offered was entry into a prize draw to win a £100 voucher for a large online company selling an extensive variety of products. The choice of the incentive reflected evidence that monetary incentives, particularly large ones, seem more effective than non-monetary incentives, which in turn appear to be more effective than no incentive in improving response [14]. Evidence also suggests that prize draws for larger monetary incentives are no less effective than small guaranteed incentives [19]. There is no evidence available to suggest what GPs perceive to be a ‘large’ incentive. GPs were not informed of their probability of winning the prize draw.

To be eligible for participation in the survey, respondents had to be fully qualified GPs and to have managed a patient with CKP in the last six months. Any recipients of the questionnaire not meeting these inclusion criteria were asked to indicate this on the front of the questionnaire and return it. These individuals were excluded from the study.

At the initial mailing, GPs were sent a personalised combined cover letter and information sheet along with their questionnaire and postage-paid reply envelope. Non-responders to this initial mailing were mailed a reminder postcard after two weeks, which was printed on A5 yellow card. Non-responders to the reminder postcard were mailed a personalised reminder letter with a second copy of the questionnaire and a postage-paid reply envelope after a further 2 weeks (i.e. 4 weeks after the first questionnaire). On the cover letter/post card at each mailing, non-responding GPs were invited to provide via post a reason for non-response (multiple response options provided and included ‘other’) and minimum data about themselves (gender, year of qualification, practice size and setting) in order to permit assessment of non-response bias [10]. Cover letters indicated the completion time of the accompanying questionnaire.

Simple descriptive statistics were used to describe the number of GPs responding to the survey and the demographic characteristics of respondents and those returning minimum data. Responses to the questionnaires were compared between the four groups using Pearson’s Chi Square test. The percentage differences in response between i) those receiving the StQ and those receiving the AbQ and ii) those offered the prize draw monetary incentive and not offered the incentive were calculated with corresponding 95% CI. The difference in response between those offered versus not offered an incentive, within each questionnaire type separately, was also examined using Pearson’s Chi Square test. Descriptive and Chi Squared analyses were performed using IBM SPSS Statistics (Version 20) and percentage difference confidence intervals were calculated using Microsoft Excel (2010).

## Results

From 800 questionnaires mailed, 19 GPs did not meet inclusion criteria, 172 completed questionnaires (adjusted response 22.0%) and 74 minimum data responses were received. Levels of response for each group were: 1) 21.8% (43 completed questionnaires out of 197, StQ with incentive), 2) 20.1% (39/194, StQ no incentive), 3) 20.1% (39/194, AbQ with incentive) and 4) 26.0% (51/196, AbQ no incentive) (Table 1). There were no statistically significant differences in response between the four groups (Chi Square value 2.661, degrees of freedom (df) 3, p = 0.447).

**Table 1:** **Response according to group**

	Incentive	No incentive	Total
**Standard questionnaire**	Group 1	Group 2	
	43/197 (21.8%)	39/194 (20.1%)	82/391 (21.0%)
**Abbreviated questionnaire**	Group 3	Group 4	
	39/194 (20.1%)	51/196 (26.0%)	90/390 (23.1%)
**Total**	82/391 (21.0%)	90/390 (23.1%)	172/781 (22.0%)

Pearson’s Chi Square value = 2.661, df 3, p = 0.447.

The majority of GPs (n = 69/74, 93.2%) providing minimum data cited ‘too little time’ as their reason for not participating in the survey. Others responded that the questionnaire was too long (n = 5, 6.8%, all sent StQ), the subject was not relevant to them (n = 2, 2.7%), and the subject was of no interest to them (n = 2, 2.7%). One GP gave the free text comment that they had not completed the questionnaire as they had no remuneration for their time. Demographic characteristics among respondents and those returning minimum data only were similar (Table 2) there a higher proportion of men returning minimum data after being sent the AbQ and an apparent trend towards a lower number of years since qualification among those completing a questionnaire compared with those providing minimum data.

**Table 2:** **Demographic characteristics of respondents and those returning minimum data only**

		StQ		AbQ	
		n (%)		n (%)	
Characteristic		Questionnaire	Minimum data	Questionnaire	Minimum data
		(n = 82)	(n = 37)	(n = 89)	(n = 37)
**Male gender (%)**		40 (48.7%)	17 (48.6%)	47 (52.8%)	27 (75.0%)
Missing data		0 (0.0%)	1 (2.7%)	0 (0.0%)	1 (2.7%)
**Mean years since qualification (SD)**		14.7 (9.8)	19.1 (8.7)	18.6 (10.9)	20.5 (9.7)
Missing data		1 (1.2%)	4 (10.8%)	2 (2.2%)	7 (18.9%)
**Mean number of GPs working in practice (SD)**		6.3 (2.9)	6.1 (3.3)	6.4 (3.4)	6.3 (2.7)
Missing data		3 (3.7%)	4 (10.8%)	0 (0.0%)	4 (10.8%)
**Practice setting**	***Urban***	46 (57.5%)	20 (55.6%)	50 (56.2%)	23 (62.2%)
	***Semi-rural***	31 (38.8%)	13 (36.1%)	26 (29.2%)	12 (32.4%)
	***Rural***	3 (3.8%)	3 (8.3%)	13 (14.6%)	2 (5.4%)
Missing data		2 (2.4%)	1 (2.7%)	0 (0.0%)	0 (0.0%)

### Effect of questionnaire length

Of the 391 eligible GPs mailed the StQ, 82 (21.0%) responded, compared with 90 of the 390 (23.1%) eligible GPs who responded to the AbQ. There was no significant difference in response between the two questionnaires (% difference -2.1% (95% CI -7.9, 3.7)).

### Effect of offering a prize draw monetary incentive

Of the 391 eligible GPs offered the incentive, 82 (21.0%) responded, compared with 90 out of 390 (23.1%) eligible GPs who were not offered an incentive. There was no significant difference in response between those offered versus not offered an incentive (% difference -2.1% (95% CI -7.9, 3.7)).

The effect on response of offering an incentive was also examined by looking at each questionnaire type separately. Response to the StQ was 43 (21.8%) among 197 eligible GPs offered an incentive and 39 (20.1%) among the 194 eligible GPs not offered an incentive (Chi Square value 0.175, df 1, p = 0.675). Response to the AbQ was 39 (20.1%) among 194 eligible GPs offered an incentive and 51 (26.0%) among the 196 eligible GPs not offered an incentive (Chi Square value 1.923, df 1, p = 0.166). Although the difference in response was larger in the AbQ (-5.9% (p = 0.166) cf. 1.7% (p = 0.675)), the difference between those who were offered an incentive and those who were not was still not significantly different.

## Discussion

This study investigated whether questionnaire length or the offer of an incentive made a difference to the response obtained to a cross-sectional postal questionnaire survey of GPs’ management of CKP. Neither questionnaire length nor offering a prize draw monetary incentive had a significant effect on response.

Response to the eight-page StQ (21.0%) in this study was only a third of the mean response among GP surveys identified in a recent review [3], however questionnaire lengths in this review were often unknown. The findings from the present study are consistent with previous GP surveys which used questionnaires of similar lengths [20–22]. However, response to the four-page AbQ was less than that which may be expected if length is judged by the number of words contained within a questionnaire (~60%) [20], and that which may be achieved by using a very short (i.e. one page) questionnaire (49%) [21]. There is no standard optimum questionnaire length [14]. In part this is due to heterogeneity in the definition of length among empirical work (number of words, pages or items or time taken for completion) [14] and because an ‘appropriate length’ is likely to differ according to the target population and topic. The lack of effect on response may indicate a non-linear relationship between response and length. There may be a threshold length at which GPs choose to respond to or not [20, 22]. If so, the lack of effect of length on response in our study could be explained by both questionnaires lying the same side of such a cut-off and/or the differential between the lengths of questionnaires being insufficient to elicit a change in response behaviour. This explanation could account for similar findings from a questionnaire study undertaken among Canadian physicians (including family physicians). No difference in response was seen between those mailed a 12-page questionnaire and those mailed a six-page version (responses of 31.7% and 31.6% respectively) [23]. The idea of a threshold was supported by a study investigating the effect of the length of reminder questionnaires on response among previously non-responding GPs. This study revealed that response among those sent a shorter four-page version (23 items) in the final reminder mailing was greater than those who were only sent the full 12-page questionnaire containing 88 items (responses of 14.8% and 7.2% respectively) [22].

Our finding that offering a prize draw monetary incentive did not influence response is not consistent with the findings of previous studies [14]. Empirical evidence suggested that a large monetary incentive should improve response compared to smaller ones and/or non-monetary incentives [14] and prize draws for substantial monetary prizes may be as effective as guaranteed smaller monetary incentives [19]. Therefore a prize draw for a single large incentive was offered to GPs in this study as this type of incentive, used in this way previously had a significant impact on response [23]. In the current study, GPs were offered entry into a prize draw where one winner would receive a gift voucher. The fact that this incentive was a prize draw and that a gift voucher, rather than cash, was offered may have reduced the incentive. Entry into a lottery is classed as a non-monetary incentive and, as such, can be less effective than a monetary incentive [14]. The incentive offered in this study was entry to a prize draw for which a winner was certain. Although this is different to being given a lottery ticket or scratch card, it may have a reduced effect compared with a guaranteed incentive. Although gift vouchers may be considered as monetary, as they provide an explicit value of currency to spend Edwards et al. referred to vouchers as being non-monetary when reviewing their impact in electronic questionnaires [14]. However, non-monetary and/or voucher incentives can improve response to surveys of health professionals and the general population [14, 24], so perception of whether the prize was monetary or non-monetary does not wholly explain the lack of difference in response identified in this study. Other explanations for the lack of impact of the incentive in this study may be that: the prize was of insufficient value in this relatively wealthy population, the GPs did not perceive the odds of winning to be high enough, as we did not communicate the size of the survey, or because entry to the prize draw was conditional on whether the GP completed the questionnaire [14, 24]. It is possible that an incentive consisting of automatic smaller financial payment to all respondents may have had more effect. However, providing meaningful automatic remuneration to a large sample of GPs to undertake a questionnaire survey may render the research impractical. Provision of incentives may introduce response bias and limit generalisability [15] although the relevance of this concern among relatively affluent GPs is unknown. A better understanding of what constitutes ‘appropriate’ remuneration and what role this actually plays in determining GPs’ involvement in research is needed to increase participation in future studies.

A key factor that may account for the lack of impact of questionnaire length and the offer of an incentive on GP survey response in this study may be time [13]; particularly as requests to participate in research are common [25] and GPs have other non-clinical duties to undertake, such as continuing professional education [26]. Similar to other work [26], in this study, most (93%) GPs providing minimum data cited ‘too little time’ as their reason for not participating in this survey. If lack of time is the key issue driving non-response among GPs, then simply offering incentives and reducing the length of a questionnaire may be insufficient to promote participation in research [12, 13]. GPs are not alone in working in time-pressured healthcare environments however similar surveys among other healthcare groups often elicit greater response. For example, responses from surveys focussing on similar topics have been reported to be 63% versus 27% and 70% versus 52% from rheumatologists versus family physicians in the UK and Canada, respectively [27, 28] and 58% from UK physiotherapists [29]. The reason for the lower response among GPs is uncertain. It is possible that many GPs are not interested in, or do not prioritise, CKP, compared with other conditions. Therefore, GPs may cite lack of time as a more socially desirable way to communicate a lack of interest in the topic, which is known to be key influence on response [1, 14, 17]. GPs may also be subject to higher numbers of requests to participate in survey research as, being generalists, their expertise is spread across a very broad range of clinical conditions and, thus, research topics. Finally cultural issues may be relevant, for example, in the secondary care environment there has been a longer tradition of empirical research activity. Empirical work examining the attitudes of German and UK GPs about their involvement in research has revealed that even when GPs felt that research was important, they were not necessarily keen to be involved and some did not view this as part of their role [26, 30]. Distrust of, and negative attitudes of GPs towards researchers, was also highlighted [26, 30]; for example, GPs were concerned that it was the researchers’, not the patients’, best interests driving the work [30].

The strengths of this study include the clear investigation of different questionnaire lengths and the offer of an incentive on questionnaire response at the same point in time and using the same clinical topic. This is important given that response to questionnaires can be impacted by level of interest the target population has in the topic [1, 14, 17] which may vary over time. Few differences were found among the characteristics of responders and those providing minimum data only. However, there is a lack of any information about GPs who did not respond at all. Therefore the degree and likely influence of non-response bias is incompletely ascertained. A potential confounder for the impact of the length of the questionnaire on response was the use of a different question format in two items on the questionnaires. These items used closed, multiple choice question in the StQ and open free text questions in the AbQ. Although use of open questions significantly reduces response compared to closed questions [14], the impact of question format among GPs is unknown. Further, this difference was only present in 2/85 questions in the StQ and 2/36 items in the AbQ and were positioned part way through the questionnaire, therefore it is unlikely that this difference will have significantly altered the GPs’ decision to respond. The confounding effect of the question format was balanced when assessing the impact of the incentive on response, as half of both the groups receiving the StQ and AbQ were offered the incentive.

Despite much work investigating strategies to improve response, successful strategies for GP surveys remain elusive [3]. Even using newer technologies to deliver surveys electronically, response remains unchanged among students and healthcare professional surveys [23, 31]. The results of this study suggest that shorter is not inevitably better and use of a prize draw monetary voucher incentive does not influence response. We propose that irrespective of the questionnaire length or the incentive offered, many GPs feel that they simply do not have the time to respond to questionnaires [9, 12]. Therefore, while solutions to this barrier are sought, in the knowledge of low response future surveys of GPs need to oversample and take measures to estimate likely non-response bias [10].

The presence of a threshold point for length influencing response needs to be formally evaluated among GPs. While doing so, critical factors determining such a threshold must be considered; for example, a higher threshold may be identified among questionnaires investigating a topic of wider interest [1, 17] and definitions of length need to be explicit. Empirical work is needed to determine whether the impact of ‘length’ differs according to how it is defined, for example, using physical length (number of words, pages or items) versus total time for completion. Qualitative work with GPs could be undertaken to establish the presence and nature of, and influences on, a threshold for response. In the absence of knowing ideal approaches to maximise response, alternative strategies for obtaining data from large samples of GPs should be considered and formally evaluated. Strategies could include assessing the value of a) using very short questionnaires (i.e. one side of A4) containing the most pertinent questions for reminder mailings [17, 22], b) distributing questionnaires at mandatory training events to enable face-to-face sampling at a time already set-aside for non-clinical work and c) staged approaches, whereby further questions are sent upon receipt of initial response. This latter strategy could be undertaken using postal, electronic or mobile phone text-based methods. Research is also needed to establish what constitutes an appropriate and meaningful incentive among GPs, particularly when time is an issue. Although UK GPs are not required to participate in research, they are expected to have a sound understanding of research methodologies, know how to appraise findings and apply results to their patients [32]. In order to sustain meaningful primary care research, work should be undertaken to establish the barriers to GPs engaging in this work and solutions to address these issues should be sought. Approaches to do so could involve establishing what constitutes appropriate remuneration [33], acknowledging that this may depend on the clinical interest of the topic being investigated, developing mechanisms by which research requests are limited to manageable numbers, although this risks being influenced by political priorities, or by including at least some level of research activity as a mandatory component for revalidation.

## Conclusions

Questionnaire length and offering a prize draw financial incentive did not affect response to a postal survey of GPs. To maximise response and thus data quality and generalisability from GP surveys, further research is needed to establish optimal questionnaire length and incentives and to systematically investigate, and seek solutions for, barriers to GP participation in research.

## Authors’ information

EC is an Academic GP. ER is a Senior Lecturer in Rheumatology and Consultant Rheumatologist, TR is a research assistant in biostatistics, ET is a biostatistician, MP is a Senior Lecturer in General Practice and NF is an academic physiotherapist and NIHR Research Professor of Musculoskeletal Health in Primary Care.

## Electronic supplementary material

Additional file 1: **Standard questionnaire with incentive.** (PDF 576 KB)

Additional file 2: **Abbreviated questionnaire with no incentive.** (PDF 315 KB)

## Abbreviations

AbQ: Abbreviated questionnaire
CI: Confidence interval
CKP: Chronic knee pain
Df: Degrees of freedom
GP: General practitioner
KOA: Knee osteoarthritis
StQ: Standard questionnaire.

## References

[1] Cartwright A (1978). Professionals as responders: variations in and effects of response rates to questionnaires, 1961–77. Br Med J.

[2] Brownell LW, Naik PC (2001). Does method of distribution improve GPs' response rate in questionniare studies?. J Epidemiol Commun H.

[3] Creavin ST, Creavin AL, Mallen CD (2011). Do GPs respond to postal questionnaire surveys? A comprehensive review of primary care literature. Fam Pract.

[4] Bishop A, Foster NE, Thomas E, Hay EM (2008). How does the self-reported clinical management of patients with low back pain relate to the attitudes and beliefs of health care practitioners? A survey of UK general practitioners and physiotherapists. Pain.

[5] Wynne-Jones G, Mallen CD, Main CJ, Dunn KM (2010). Sickness certification and the GP: what really happens in practice?. Fam Pract.

[6] Clarson L (2011). Do general practitioners monitor patients with osteoarthritis?. Ann Rheum Dis.

[7] Cockburn J, Campbell E, Gordon JJ, Sanson-Fisher RW (1988). Response bias in a study of general practice. Fam Pract.

[8] Stocks N, Gunnell D (2000). What are the characteristics of general practitioners who routinely do not return postal questionnaires: a cross sectional study. J Epidemiol Community Health.

[9] Barclay S, Todd C, Finley I, Grande G, Wyatt P (2002). Not another questionnaire! Maximising the response rate, predicting non-response and assessing non-response bias in postal questionnaire studies of GPs. Fam Pract.

[10] de Vaus D (2014). Chapter 6: finding a sample. Surveys in Social Research.

[11] Sibbald B, Addington-Hall J, Brenneman D, Freeling P (1994). Telephone versus postal surveys of general practitioners: methodological considerations. Br J Gen Pract.

[12] Kaner EFS, Haighton CA, McAvoy BR (1998). "So much post, so busy with practice - so, no time!": a telephone survey of general practitioners' reasons for not participating in postal questionniare surveys. BJGP.

[13] Armstrong D, Ashworth M (2000). When questionnaire response rates do matter: a survey of general practitioners and their views of NHS changes. Br J Gen Pract.

[14] Edwards PJ, Roberts I, Clarke MJ, DiGuiseppi C, Wentz R, Kwan I (2009). Methods to increase response to postal and electronic questionnaires (Review). The Cochrane database of systematic reviews.

[15] Draper H, Wilson S, Flanagan S, Ives J (2009). Offering payments, reimbursement and incentives to patients and family doctors to encourage participation in research. Fam Pract.

[16] Cottrill M (1996). Surveys demand too much time.

[17] Sim J, Wright C (2000). Research in health care.

[18] de Vaus D (2014). Chapter 7: constructing questionnaires. Surveys in Social Research.

[19] Brueton VC, Tierney JF, Stenning S, Meredith S, Harding S, Nazareth I (2014). Strategies to improve retention in randomised trials: a Cochrane systematic review and meta-analysis. BMJ Open.

[20] Jepson C, Asch DA, Hershey JC, Ubel PA (2005). In a mailed physician survey, questionnaire length had a threshold effect on response rate. J Clin Epidemiol.

[21] Kowall B, Breckenkamp J, Heyer K, Berg-Beckhoff G (2010). German wide cross sectional survey on health impacts of electromagnetic fields in the view of general practitioners. Int J Pub Health.

[22] Glidewell L, Thomas R, MacLennan G, Bonetti D, Johnston M, Eccles MP (2012). Do incentives, reminders or reduced burden improve healthcare professional response rates in postal questionnaires? Two randomised controlled trials. BMC Health Serv Res.

[23] Grava-Gubins I, Scott S (2008). Effects of various methodologic strategies. Survey response rates among Canadian physicians and physicians in training. Can Fam Phys.

[24] Thorpe C, Ryan B, McLean SL, Burt A, Stewart M, Brown JB (2009). How to obtain excellent response rates when surveying physicians. Fam Pract.

[25] Moore M, Post K, Smith H (1999). 'Bin bag' study: a survey of the research requests received by general practitioners and the primary health care team. Br J Gen Pract.

[26] Hummers-Pradier E, Scheidt-Nave C, Martin H, Heinemann S, Kochen MM, Himmel W (2008). Simply no time? Barriers to GPs' participation in primary health care research. Fam Pract.

[27] Coyte PC, Hawker G, Croxford R, Attard C, Wright JG (1996). Variation in rheumatologists and family physicians' perceptions of the indications for an outcomes of knee replacement surgery. J Rheumatol.

[28] Chard J, Dickson J, Tallon D, Dieppe P (2002). A comparison of the views of rheumatologists, general practitioners and patients on the treatment of osteoarthritis. Rheumatology.

[29] Holden MA, Nicholls EE, Hay EM, Foster NE (2008). Physical therapists' use of therapeutic exercise for patients with clinical knee osteoarthritis in the United Kingdom: in line with current recommendations?. Phys Ther.

[30] Mason V, Shaw A, Wiles N, Mulligan J, Peters T, Sharp D (2007). GPs' experiences of primary care mental health research: a qualitative study of the barriers to recruitment. Fam Pract.

[31] Kaplowitz MD, Hadlock TD, Levine R (2004). A comparison of web and mail survey response rates. Public Opin Quart.

[32] Royal College of General Practitioners (2013). Royal College of General Practitioners Curriculum 2010: 2.04 The contextual statement on enhancing professional knowledge.

[33] Cadwallader JS, Lebeau JP, Lasserre E, Letrilliart L (2014). Patient and professional attitudes towards research in general practice: the RepR qualitative study. BMC Fam Pract.

[CR34] The pre-publication history for this paper can be accessed here:http://www.biomedcentral.com/1471-2288/15/3/prepub

