# Supplementary material for: Maximising response from GPs to questionnaire surveys: do length or incentives make a difference?
Source: BMC Med Res Methodol. 2015 Jan 6;15:3. doi: 10.1186/1471-2288-15-3 (PMC4293861; doi:10.1186/1471-2288-15-3)
Supplement: Supplementary file 1 — Additional file 1: Standard questionnaire with incentive. (PDF 576 KB) [file 12874_2014_1152_MOESM1_ESM.pdf]

Keele University

Unique survey ID	

Management of Chronic Knee Pain Study

We are seeking the views of general practitioners who treat patients over 45 years old, with chronic knee pain.

If you **have not** managed someone with chronic knee pain in the last 6 months, please tick this box and return the questionnaire without completing it any further. ☐

If you **are not** a general practitioner, please tick this box and return the questionnaire without completing it any further ☐

This questionnaire should take no more than **15 minutes** to complete.

Return of your completed questionnaire will be interpreted as you providing your consent to participate in this study.

Once you have completed your questionnaire please return it to Dr Elizabeth Cottrell, Academic Clinical Fellow GP Specialty Trainee, at the Arthritis Research UK Primary Care Centre, Keele University, Staffordshire, ST5 5BG using the enclosed FREEPOST envelope.

If you have any questions about this questionnaire or the study in general you can email Elizabeth Cottrell at e.cottrell@cphc.keele.ac.uk

Instructions for completing this questionnaire

- ✓ When completing the questionnaire, please try and be as honest as possible throughout. There are no ‘correct’ or ‘incorrect’ answers.
- ✓ Where relevant please answer questions by ticking a box e.g. ☑
- ✓ We are interested in your clinical opinion about patients **aged 45 years and over** with **chronic knee pain**. In this age group chronic knee pain is synonymous with knee osteoarthritis.
- ✓ Answer all questions using the definition of chronic knee pain as follows: knee pain and associated symptoms that have been present for more than 3 months and does not result from a fracture, infection, systemic rheumatological problem, metastases or surgery.
- ✓ Please do not consult any literature while completing this questionnaire.
- ✓ If you wish to enter the prize draw to win £100 worth of Amazon vouchers, please complete your details at the back of the questionnaire.

Thank you for your help with this study

REC Number: XX/XXXXX/XX

Version number, Date:

Section 1: About you

1.1 Please state the year in which you qualified as a General Practitioner ☐

1.2 How many General Practitioners work in your practice (including yourself)? ☐

1.3 How do you best **describe yourself** (please tick **one box** only)

☐ GP Partner ☐ Salaried GP ☐ Locum GP

☐ GP trainer ☐ Other

If you ticked '**other'**, please specify

____

1.4 Is your practice ☐ Urban? ☐ Semi-rural? ☐ Rural?

1.5 Are you ☐ Male? ☐ Female?

1.6 Are you a **GP with a special interest (GPwSI)** in musculoskeletal conditions?

☐ No ☐ Yes

1.7 Do you remember receiving any **specific undergraduate training** in the field of chronic knee pain?

☐ No ☐ Yes ☐ Don't know or cannot remember

If yes, please give details:

1.8 Do you remember receiving any **specific postgraduate training** in the field of chronic knee pain?

☐ No ☐ Yes ☐ Don't know or cannot remember

If yes, please give details:

1.9 Do you have, or have you ever suffered from chronic knee pain **yourself**?

☐ No ☐ Yes

Section 2: Your views about chronic knee pain

Below is a list of **possible causes** for a patient developing chronic knee pain. Please indicate the extent to which you agree or disagree with these causes by ticking one box in each row.

		Strongly disagree	Disagree	Neither agree or disagree	Agree	Strongly agree
**2.1**	Hereditary/runs in the family					
**2.2**	Being overweight/obese					
**2.3**	A person's own mental attitude e.g. thinking about life negatively					
**2.4**	A person's emotional state e.g. feeling down, anxious					
**2.5**	Ageing					
**2.6**	Accident or injury					
**2.7**	Manual work					
**2.8**	Sport					
**2.9**	Osteoarthritis					
**2.10**	Changes consistent with osteoarthritis seen on x-ray					

Please consider the **GPs role** in managing patients aged over 45 years old with chronic knee pain. Please indicate the extent to which you agree or disagree with the statements given by ticking one box per row.

		Strongly disagree	Disagree	Neither agree or disagree	Agree	Strongly agree
**2.11**	It is part of a GP's job to manage people with chronic knee pain					
**2.12**	GPs have enough time to manage patients with chronic knee pain					
**2.13**	Managing patients with chronic knee pain is a priority for GPs					
**2.14**	Managing patients with chronic knee pain is of clinical interest to me					

When completing the following questions, please consider patients aged over 45 years old with **chronic knee pain**. Please indicate the extent to which you agree or disagree with the statements given by ticking one box per row.

		Strongly disagree	Disagree	Neither agree or disagree	Agree	Strongly agree
**2.15**	Mental stress can cause chronic knee problems even in the absence of tissue damage					
**2.16**	The cause of chronic knee problems is unknown					
**2.17**	Pain is a nociceptive stimulus, indicating tissue damage					
**2.18**	A patient suffering from a severe chronic knee problem will benefit from physical exercise					
**2.19**	Functional limitations associated with chronic knee problems are the result of psychosocial factors					
**2.20**	Patients with chronic knee pain should preferably practise only pain free movements					
**2.21**	Therapy may have been successful even if pain remains					
**2.22**	Chronic knee pain indicates the presence of organic injury					
**2.23**	If chronic knee pain increases in severity, I immediately adjust the intensity of my treatment accordingly					
**2.24**	If therapy does not result in a reduction in chronic knee pain, there is a high risk of severe restrictions in the long term					
**2.25**	Pain reduction is a precondition for the restoration of normal functioning					
**2.26**	Increased pain indicates new tissue damage or the spread of existing damage					
**2.27**	There is no effective treatment to eliminate chronic knee problems					
**2.28**	Even if the pain has worsened, the intensity of the next treatment can be increased					
**2.29**	If patients complain of pain during exercise, I worry that damage is being caused					
**2.30**	The severity of tissue damage determines the level of pain					
**2.31**	Learning to cope with stress promotes recovery from chronic knee problems					
**2.32**	Exercises that may be knee straining should not be avoided					
**2.33**	In the long run, patients with chronic knee pain have a higher risk of developing severe functional impairments					

Section 3: Clinical scenario of a patient with chronic knee pain

Presented below is a clinical scenario of a patient **with chronic knee pain** that presents to you with this problem for the first time. All questions that follow relate to the care you would give this particular patient. Think about the patient's first consultation with you.

Patient: Mrs Jones, 58-year-old Prison Officer

History:
First presentation of gradually worsening bilateral knee pain (right worse than left) over 2 years
No history of trauma
Pain always present when walking and at rest, worst when climbing stairs. No night pain.
Managing activities of daily living. Difficulty gardening.
Stopped going to gym – thinks was making pain worse
Only treatment tried is ibuprofen once or twice when pain "really bad" – no benefit.
Came today finding work increasingly difficult due to the stairs
Usually well – no comorbidities

Medication: Nil

Examination:
Body Mass Index 33
Knees – bilaterally no effusions. Joint tenderness upon palpation. Bilateral coarse crepitations.
Slightly reduced flexion of the right knee.
Hips – no abnormality detected

3.1 The patient's **symptoms** are: (please tick the **one** box that best reflects your opinion)

☐ Very severe ☐ Severe ☐ Moderate ☐ Mild ☐ Very mild

3.2 It is most likely that this patient's symptoms result from **knee damage** that is: (please tick the **one** box that best reflects your opinion)

☐ Very severe ☐ Severe ☐ Moderate ☐ Mild ☐ Very mild

3.3 What **investigations** will you do/order for this patient at this point? (please tick **all** that apply)

☐ None
☐ Lab test (e.g. inflammatory markers)
☐ Special imaging (e.g. CT, MRI, myelogram, bone scan)
☐ Knee x-ray
☐ X-ray of other area
☐ Synovial fluid aspirate/analysis
☐ Other

If you ticked '**x-ray of other area'** or **'other'** please specify**:**

____

3.4 Would you **refer** this patient to see someone else at this point? ☐ Yes ☐ No

If yes, to whom would you refer her?

____

____

____

3.5 What **diagnosis** would you give to this patient at this point?

____

3.6 Using the words you would use with the patient, ***briefly*** state how you would **describe your diagnosis** to the patient?

____

____

____

3.7 Using the words you would use with the patient, ***briefly*** describe **what the future is likely to hold** with regards to her knee problem?

____

____

3.8 **At this consultation**, what approaches would you use or suggest to manage this patient? (please tick **all** that apply)

☐ Non-selective NSAIDs	☐ Paracetamol	☐ Opiates
☐ COX II inhibitor	☐ Anti-depressants	☐ Topical NSAID preparation
☐ Injection of hyaluronan	☐ Injection of steroids	☐ Topical capsaicin preparation
☐ Insoles	☐ Heat	☐ Transcutaneous electrical nerve stimulation
☐ Rest	☐ Bed rest	☐ Quadriceps strengthening exercises
☐ General exercise	☐ Ice	☐ Provision of walking stick(s)
☐ Acupuncture	☐ Other	☐ None

If you ticked **'other'** please specify

____

3.9 Do you usually provide **written information** for patients in this situation? ☐ Yes ☐ No

If yes, please give details (e.g. name of and/or source of booklet/leaflet, website address)

____

3.10 Would you offer any **other advice** as part of your treatment ☐ Yes ☐ No

If yes, please state the nature of your advice

____

____

If you ticked an answer involving "Exercise" in question 3.8 please go on to answer questions 3.11-3.13 below. If not, please move straight on to Section 4

3.11 What **kind of exercise** would you suggest to this patient at this stage?

____

3.12 Using the words you would use with the patient, briefly state what **advice** you would give regarding exercise at this stage

____

____

3.13 Would you **check** if this patient was completing her exercise programme? ☐ Yes ☐ No

If yes, please specify how would you do this?

____

____

Section 4: Your views about the role of exercise in treating chronic knee pain

We are interested in your views about the **role of exercise** in the treatment of **chronic knee pain in patients over 45 years old**. Please indicate the extent to which you agree or disagree with the statements given by ticking one box per row.

		Strongly disagree	Disagree	Neither agree or disagree	Agree	Strongly agree
4.1	GPs should prescribe quadriceps strengthening exercises to every patient with chronic knee pain					
4.2	GPs should prescribe general exercise, for example, walking or swimming, for every patient with chronic knee pain					
4.3	Knee problems are improved by quadriceps strengthening exercises					
4.4	Knee problems are improved by general exercise, for example walking or swimming					
4.5	Quadriceps strengthening exercises for the knee are safe for everybody to do					
4.6	General exercise, for example walking or swimming is safe for everybody to do					
4.7	Exercise for chronic knee pain is most beneficial when it is tailored to meet individual patient needs					
4.8	A standard set of exercises is sufficient for every patient with chronic knee problems					
4.9	GPs should educate chronic knee pain patients about how to change their lifestyle for the better					
4.10	It is important that people with chronic knee pain increase their overall activity levels					
4.11	How well a patient complies with their exercise programme determines how effective it will be					
4.12	GPs should follow up patients to monitor extent of continuation of exercises					
4.13	It is the patient's own responsibility to continue doing their exercise programme					
4.14	Exercise is effective for patients if an x-ray shows severe knee osteoarthritis					
4.15	Exercise works just as well for everybody, regardless of the amount of pain they have					
4.16	Increasing the strength of the muscles around the knee stops the knee problem getting worse					
4.17	Increasing overall activity levels stops the knee problem getting worse					
4.18	Exercise for chronic knee pain is only effectively provided by physiotherapists					
4.19	Time constraints prevent GPs from providing advice on individual exercises for chronic knee pain					
4.20	Exercise for chronic knee pain should only be used after drug treatment has been tried					
4.21	Exercise for chronic knee pain would be used more frequently if access to physiotherapy was easier					

4.22 What do you feel is your role as a GP in exercise as a treatment for chronic knee pain?

____

____

____

____

Section 5: Guidelines

Please indicate the degree to which you are familiar with the current **NICE guidelines**.

		I have never heard about or read them	I have heard of them but not seen them	I have seen them but not read them	I have read the full guidance and/or summary	I consider the guidance when planning management
**5.1**	How much have you heard or read about the guideline published by NICE in 2008 for the care and management of osteoarthritis in adults?					

We are interested to know how much you value **NICE guidelines** as a tool to inform your clinical practice. Please indicate the degree to which you agree with the following statements by ticking one box per row.

		Strongly disagree	Disagree	Neither agree or disagree	Agree	Strongly agree
**5.2**	NICE is a credible source of guidance					
**5.3**	NICE guidelines are primarily targeted at GPs					
**5.4**	NICE guidelines are primarily targeted at secondary care					
**5.5**	NICE guidelines are primarily targeted at allied health professionals					
**5.6**	NICE guidelines are easily implemented in real-life situations					
**5.7**	NICE guidelines improve my management of patients					

**5.8**	We are interested to hear about **your experiences of implementing guidelines** in the management of chronic knee pain in the GP setting. Please describe any guidelines you find particularly helpful or relevant positive experiences, concerns about, barriers to use or memorable events regarding using guidelines for managing chronic knee pain<br>____<br>____<br>____<br>____

Would you be happy for us to contact you again in the future regarding this study?

☐ Yes ☐ No

Would you like to be entered into the prize draw to win £100 worth of Amazon vouchers?

☐ Yes ☐ No

If you answered YES to either of the questions above please provide your name and contact details below (these details will be kept separately from your responses to the questionnaire):

**Name:**	
**Daytime telephone number:**	
**Address:**	
**Email**	

End of Questionnaire

You have reached the end of the questionnaire. Please return the questionnaire in the **FREEPOST** envelope provided.

If you have any questions about this questionnaire or the study in general, you can email Elizabeth Cottrell at e.cottrell@cphc.keele.ac.uk

Thank you for taking the time to complete this questionnaire. Your time and participation is greatly appreciated.

Unique survey ID
