# Supplementary material for: Maximising response from GPs to questionnaire surveys: do length or incentives make a difference?
Source: BMC Med Res Methodol. 2015 Jan 6;15:3. doi: 10.1186/1471-2288-15-3 (PMC4293861; doi:10.1186/1471-2288-15-3)
Supplement: Supplementary file 2 — Additional file 2: Abbreviated questionnaire with no incentive. (PDF 315 KB) [file 12874_2014_1152_MOESM2_ESM.pdf]

Unique survey ID	

Management of Chronic Knee Pain Study

We are seeking the views of general practitioners who treat patients over 45 years old with chronic knee pain.

If you **have not** managed someone with chronic knee pain in the last 6 months, please tick this box and return the questionnaire without completing it any further. ☐

If you **are not** a general practitioner, please tick this box and return the questionnaire without completing it any further ☐

This questionnaire should take no more than **10 minutes** to complete.

Return of your completed questionnaire will be interpreted as you providing your consent to participate in this study.

Once you have completed your questionnaire please return it to Dr Elizabeth Cottrell, Academic Clinical Fellow GP Specialty Trainee, at the Arthritis Research UK Primary Care Centre, Keele University, Staffordshire, ST5 5BG using the enclosed FREEPOST envelope.

If you have any questions about this questionnaire or the study in general you can email Elizabeth Cottrell at e.cottrell@cphc.keele.ac.uk

Instructions for completing this questionnaire

- ✓ When completing the questionnaire, please try and be as honest as possible throughout. There are no ‘correct’ or ‘incorrect’ answers.
- ✓ Where relevant please answer questions by ticking a box e.g. ☑
- ✓ We are interested in your clinical opinion about patients **aged 45 years and over** with **chronic knee pain**. In this age group, chronic knee pain is synonymous with knee osteoarthritis.
- ✓ Answer all questions using the definition of chronic knee pain as follows: knee pain and associated symptoms that have been present for more than 3 months and does not result from a fracture, infection, systemic rheumatological problem, metastases or surgery.
- ✓ Please do not consult any literature while completing this questionnaire.

Thank you for your help with this study

REC Number: XX/XXXXX/XX

Version number, Date:

Section 1: About you

**1.1**	Please state the year in which you qualified as a General Practitioner	☐
**1.2**	How many General Practitioners work in your practice (including yourself)?	☐

1.3 Is your practice ☐ Urban? ☐ Semi-rural? ☐ Rural?

1.4 Are you ☐ Male? ☐ Female?

We are interested in your familiarity with and views on **guidelines for chronic knee pain.** Please indicate the answer that best applies to you by ticking one box per row.

		I have never heard of them	I have heard of them but not seen them	I have seen them but not read them	I have read the full guidance and/or summary	I consider the guidance when planning management
**1.5**	How much have you heard or read about the guideline published by NICE in 2008 for the care and management of osteoarthritis in adults?					

		Strongly disagree	Disagree	Neither agree or disagree	Agree	Strongly agree
**1.6**	NICE is a credible source of guidance					

Please consider the **GPs role** in managing patients aged over 45 years old with chronic knee pain. Please indicate the extent to which you agree or disagree with the statements given by ticking one box per row.

		Strongly disagree	Disagree	Neither agree or disagree	Agree	Strongly agree
**1.7**	It is part of a GP's job to manage people with chronic knee pain					
**1.8**	GPs have enough time to manage patients with chronic knee pain					
**1.9**	Managing patients with chronic knee pain is a priority for GPs					
**1.10**	Managing patients with chronic knee pain is of clinical interest to me					

Section 2: Clinical scenario of a patient with chronic knee pain

Presented below is a clinical scenario of a patient **with chronic knee pain** that presents to you with this problem for the first time. All questions that follow relate to the care you would give this particular patient. Think about the patient's first consultation with you.

Patient: Mrs Jones, 58-year-old Prison Officer

History: First presentation of gradually worsening bilateral knee pain (right worse than left) over 2 years
No history of trauma
Pain always present when walking and at rest, worst when climbing stairs. No night pain.
Managing activities of daily living. Difficulty gardening.
Stopped going to gym – thinks was making pain worse
Only treatment tried is ibuprofen once or twice when pain "really bad" – no benefit.
Came today finding work increasingly difficult due to the stairs
Usually well – no comorbidities

Medication: Nil

Examination: Body Mass Index 33
Knees – bilaterally no effusions. Joint tenderness upon palpation. Bilateral coarse crepitations. Slightly reduced flexion of the right knee.
Hips – no abnormality detected

2.1 What (if any) **investigations** will you do/order for this patient at this point? (if none please write "**None**")

____

____

____

2.2 Would you **refer** this patient to see someone else at this point? ☐ Yes ☐ No

If yes, to whom would you refer her?

____

____

____

2.3 What **diagnosis** would you give to this patient at this point?

____

2.4 **At this consultation**, what approaches would you use or suggest to manage this patient?

____

____

____

____

____

____

Section 3: Your views about the role of exercise in treating chronic knee pain

We are interested in your views about the **role of exercise** in the treatment of **chronic knee pain in patients over 45 years old**. Please indicate the extent to which you agree or disagree with the statements given by ticking one box per row.

		Strongly disagree	Disagree	Neither agree or disagree	Agree	Strongly agree
**3.1**	GPs should prescribe quadriceps strengthening exercises to every patient with chronic knee pain					
**3.2**	GPs should prescribe general exercise, for example, walking or swimming, for every patient with chronic knee pain					
**3.3**	Knee problems are improved by quadriceps strengthening exercises					
**3.4**	Knee problems are improved by general exercise, for example walking or swimming					
**3.5**	Quadriceps strengthening exercises for the knee are safe for everybody to do					
**3.6**	General exercise, for example walking or swimming is safe for everybody to do					
**3.7**	Exercise for chronic knee pain is most beneficial when it is tailored to meet individual patient needs					
**3.8**	A standard set of exercises is sufficient for every patient with chronic knee problems					
**3.9**	GPs should educate chronic knee pain patients about how to change their lifestyle for the better					
**3.10**	It is important that people with chronic knee pain increase their overall activity levels					
**3.11**	How well a patient complies with their exercise programme determines how effective it will be					
**3.12**	GPs should follow up patients to monitor extent of continuation of exercises					
**3.13**	It is the patient's own responsibility to continue doing their exercise programme					
**3.14**	Exercise is effective for patients if an x-ray shows severe knee osteoarthritis					
**3.15**	Exercise works just as well for everybody, regardless of the amount of pain they have					
**3.16**	Increasing the strength of the muscles around the knee stops the knee problem getting worse					
**3.17**	Increasing overall activity levels stops the knee problem getting worse					
**3.18**	Exercise for chronic knee pain is only effectively provided by physiotherapists					
**3.19**	Time constraints prevent GPs from providing advice on individual exercises for chronic knee pain					
**3.20**	Exercise for chronic knee pain should only be used after drug treatment has been tried					
**3.21**	Exercise for chronic knee pain would be used more frequently if access to physiotherapy was easier					

**3.22**	What do you feel is your role as a GP in exercise as a treatment for chronic knee pain?<br>____<br>____<br>____<br>____

Would you be happy for us to contact you again in the future regarding this study?

☐ Yes ☐ No

If you answered YES to the question above please provide your name and contact details below (these details will be kept separately from your responses to the questionnaire):

**Name:**	
**Daytime telephone number:**	
**Address:**	
**Email**	

End of Questionnaire

You have reached the end of the questionnaire. Please return the questionnaire in the **FREEPOST** envelope provided.

If you have any questions about this questionnaire or the study in general, you can email Elizabeth Cottrell at e.cottrell@cphc.keele.ac.uk

Thank you for taking the time to complete this questionnaire. Your time and participation is greatly appreciated.

Unique survey ID
